# Structure, Dynamics, Receptor Binding, and Antibody Binding of the Fully Glycosylated Full-Length SARS-CoV-2 Spike Protein in a Viral Membrane

**DOI:** 10.1021/acs.jctc.0c01144

**Published:** 2021-03-10

**Authors:** Yeol Kyo Choi, Yiwei Cao, Martin Frank, Hyeonuk Woo, Sang-Jun Park, Min Sun Yeom, Tristan I. Croll, Chaok Seok, Wonpil Im

**Affiliations:** † Departments of Biological Sciences, Chemistry, Bioengineering, and Computer Science and Engineering, Lehigh University, Bethlehem, Pennsylvania 18015, United States; ‡ Biognos AB, Box 8963, 40274 Göteborg, Sweden; § Department of Chemistry, Seoul National University, Seoul 08826, Republic of Korea; ∥ Korean Institute of Science and Technology Information, Daejeon 34141, Republic of Korea; ⊥ Department of Haematology, Cambridge Institute for Medical Research, University of Cambridge, Cambridge CB2 0XY, U.K.

## Abstract

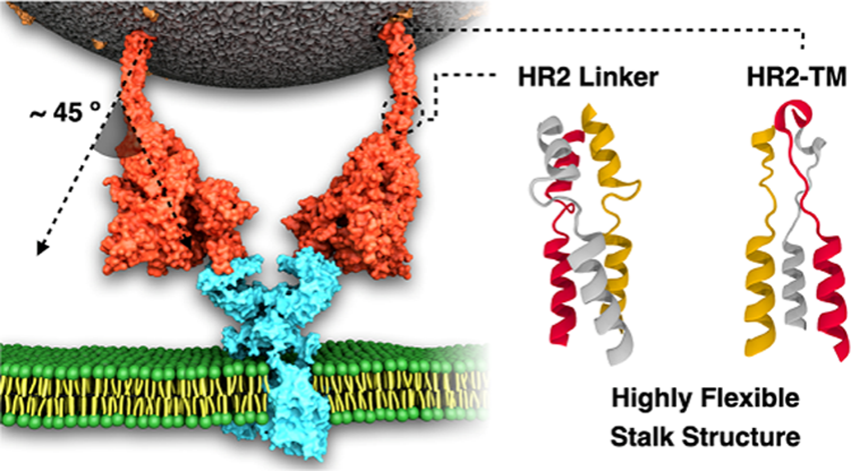

The spike (S) protein of severe acute respiratory syndrome coronavirus 2 (SARS-CoV-2) mediates host cell entry by binding to angiotensin-converting enzyme 2 (ACE2) and is considered the major target for drug and vaccine development. We previously built fully glycosylated full-length SARS-CoV-2 S protein models in a viral membrane including both open and closed conformations of the receptor-binding domain (RBD) and different templates for the stalk region. In this work, multiple μs-long all-atom molecular dynamics simulations were performed to provide deeper insights into the structure and dynamics of S protein and glycan functions. Our simulations reveal that the highly flexible stalk is composed of two independent joints and most probable S protein orientations are competent for ACE2 binding. We identify multiple glycans stabilizing the open and/or closed states of the RBD and demonstrate that the exposure of antibody epitopes can be captured by detailed antibody–glycan clash analysis instead of commonly used accessible surface area analysis that tends to overestimate the impact of glycan shielding and neglect possible detailed interactions between glycan and antibodies. Overall, our observations offer structural and dynamic insights into the SARS-CoV-2 S protein and potentialize for guiding the design of effective antiviral therapeutics.

## 1 Introduction

The outbreak of Coronavirus disease 2019 (COVID-19) caused by severe acute respiratory syndrome coronavirus 2 (SARS-CoV-2) presents a tremendous threat to global public health. It caused over 35 million confirmed cases and more than 1 million deaths as of October, 2020. Due to the unavailability of antiviral medicines or approved vaccines, the current treatment strategy is supportive care to relieve symptoms and isolation of infected individuals to reduce transmission, which has placed a huge burden on the public healthcare system and led to massive social and economic distress.

SARS-CoV-2 is an enveloped virus with a positive-sense single-stranded RNA genome.^1^ The spike (S) protein anchored in the viral envelope is a class I fusion protein that mediates receptor binding and host cell entry by interacting with human angiotensin-converting enzyme 2 (ACE2),^2−4^ and it is also the target of a variety of neutralizing antibodies.^5−8^ The S protein is a homotrimer and each monomer has two subunits (S1 and S2) separated by a cleavage site that is recognized by host proteases.^9^ A number of recently published structural studies using cryogenic electron microscopy (cryo-EM) have provided a good understanding of the S protein structure at near-atomic resolution.^4,10−12^ The S1 subunit responsible for receptor binding is composed of the signal peptide (SP), N terminal domain (NTD), and receptor-binding domain (RBD), and the S2 subunit responsible for membrane fusion is composed of the fusion peptide (FP), two heptad repeats (HR1 and HR2), transmembrane domain (TM), and cytoplasmic domain (CP). The three RBDs on the top of the S protein head are conformationally variable. In closed conformations, all three RBDs lay flat with the receptor-binding motif occluded by the RBDs on the neighboring monomers. In open conformations, one or multiple RBDs lift up and expose the receptor-binding motif(s).

Although the cryo-EM structures of the S protein have provided crucial information about its overall structure, highly flexible protein regions such as loops and stalk still remain unresolved. Molecular dynamics (MD) simulations provide molecular-level insights into the underlying mechanisms of biological functions that are difficult to elucidate only with experiments. Recently, cryo-electron tomography (cryo-ET) and MD simulations have been used to explore the conformational variability of the S protein stalk that gives the head orientational freedom and allows the S protein to scan the host cell surface.^13,14^ However, it still remains unclear what structural freedoms in the stalk portion are determinant to the overall shape of the S protein and its orientation, and how they affect the binding to ACE2. In addition, MD simulations along with accessible surface area (ASA) calculations have been used to estimate the impact of glycan shielding on the exposure of antibody epitopes.^15^ Mutations of two glycosylation sites have been performed to study the role of two N-linked glycans in stabilizing an RBD open conformation.^16^ Further investigation is still required to evaluate whether the ASA difference between glycosylated and non-glycosylated structures truly reflects the impact of glycan shielding on antibody binding and whether glycans have more functional roles than stabilizing the open-state RBD.

In this work, we present all-atom MD simulations of the fully glycosylated full-length S protein in a viral bilayer, and multiple μs-long trajectories were generated for the RBD in open and closed states and the S stalk built from different models. We also performed multiple μs-long simulations of non-glycosylated S head-only systems. Our results provide deeper insights into the functional roles of glycans that not only provide shielding for immune evasion but also contribute to the trimer stability and transition of RBD open and close states. Moreover, our simulations give insights into the essential structural roles of highly flexible stalk conformations in the S protein binding to ACE2.

## 2 Methods

### 2.1 Model Structures of the Fully Glycosylated Full-Length SARS-CoV-2 S Protein

**Figure 1:**
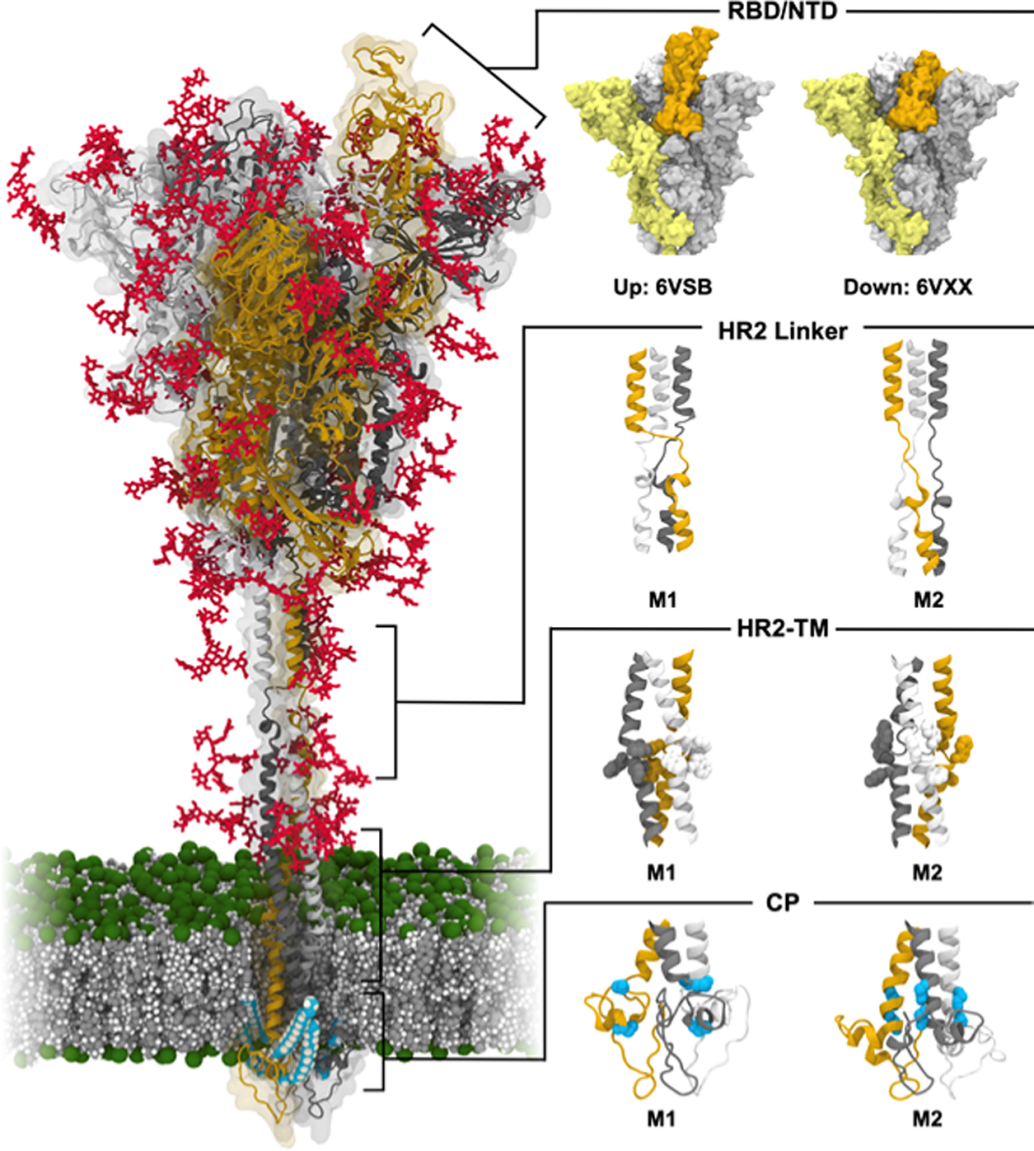
Model structure of the fully glycosylated full-length SARS-CoV-2 S protein in a viral membrane. A model structure of the SARS-CoV-2 S protein is shown on the left panel. Two models for RBD/NTD, HR2 linker, HR2-TM, and CP are enlarged on the right panel. The three individual chains of the S protein are colored in yellow, gray, and white, respectively, while glycans are represented as red sticks. The palmitoylation sites of the S protein are highlighted in cyan. The phosphate, carbon, and hydrogen atoms of the viral membrane are colored in green, gray, and white, respectively. For clarity, water molecules and ions are omitted. All illustrations were created using visual molecular dynamics (VMD).^30^

An illustrative snapshot of a fully glycosylated full-length S protein structure in a viral membrane is shown in Figure 1. When there are many missing residues and domains, the initial models for MD simulations need to be carefully built and validated against available experimental data. We have built the models using GALAXY protein modeling suite^17−19^ for missing residues and domains, ISOLDE^20^ for refining initial models against experimental density maps, and CHARMM-GUI Glycan Reader and Modeler,^21−23^ and Membrane Builder^24−26^ for glycan and membrane building. The head of the S trimer was built based on cryo-EM structures (PDB ids: 6VSB(4) and 6VXX(12)). All three chains of 6VXX have the RBD in a closed conformation. One chain of 6VSB (A chain) has the RBD in an open conformation and the other two chains have the RBD in a closed conformation. Two models were selected for each of HR2 linker, HR2-TM, and CP, resulting in a total of 16 structures after the domain by domain assembly. The glycan sequences selected for 22 N-linked and 1 O-linked glycosylation sites of each monomer were based on the mass spectrometry data.^27,28^ The detailed model generation is described in ref (29). The model name follows the model numbers used for HR2 linker, HR2-TM, and CP structures. For example, “6VSB_1_2_1” represents a model based on 6VSB with HR2 linker model 1 (M1), HR2-TM model 2 (M2), and CP model 1 (M1). All 16 S protein simulation systems and trajectories are available in CHARMM-GUI COVID-19 archive (http://www.charmm-gui.org/docs/archive/covid19).

### 2.2 Simulation Details

In this study, the CHARMM36(m) force field was used for proteins,^31^ lipids,^32,33^ and carbohydrates.^34−36^ The TIP3P water model^37^ was utilized along with a 0.15 M KCl solution. The total number of atoms is 2 343 394 (6VSB_1_1_1: 668 899 water molecules, 2128 K^+^, and 1857 Cl^–^); see CHARMM-GUI COVID-19 archive for other system information. The van der Waals interactions were smoothly switched off over 10–12 Å by a force-based switching function^38^ and the long-range electrostatic interactions were calculated by the particle-mesh Ewald method^39^ with a mesh size of ∼1 Å.

All simulations were performed using the inputs generated by CHARMM-GUI^40^ and GROMACS 2018.6^41^ for both equilibration and production with the LINCS algorithm.^42^ The temperature was maintained using a Nosé–Hoover temperature coupling method^43,44^ with a τ_t_ of 1 ps; for pressure coupling (1 bar), semi-isotropic Parrinello–Rahman method^45,46^ with a τ_p_ of 5 ps and a compressibility of 4.5 ×10^–5^ bar^–1^ was used. During the equilibration run, NVT (constant particle number, volume, and temperature) dynamics was first applied with a 1 fs time step for 250 ps. Subsequently, the NPT (constant particle number, pressure, and temperature) ensemble was applied with a 1 fs time step (for 2 ns) and with a 2 fs time step (for 18 ns). During the equilibration, positional and dihedral restraint potentials were applied, and their force constants were gradually reduced. The production run was performed with a 4 fs time step using the hydrogen mass repartitioning technique^47^ without any restraint potential. Each system ran about 20 ns/day with 1024 CPU cores on NURION in the Korea Institute of Science and Technology Information.

## 3 Results and Discussion

### 3.1 Stalk of the S Protein Consists of Two Highly Flexible Linkers

**Figure 2:**
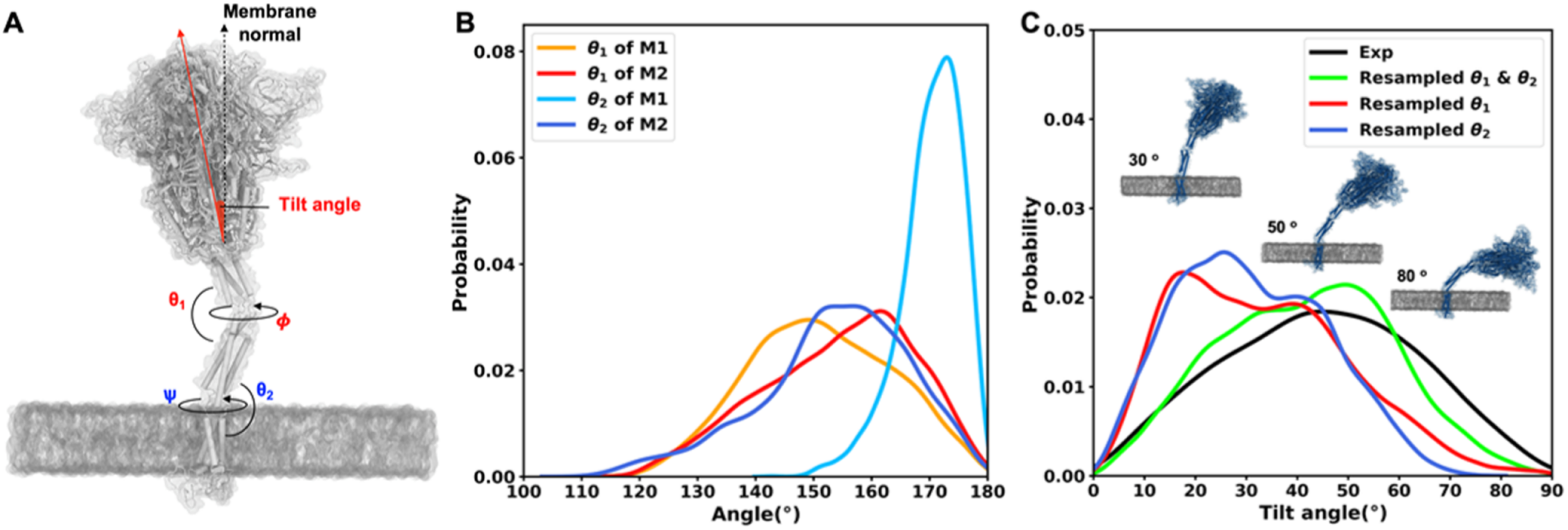
Bending motions of the S protein in a viral membrane. (A) Illustrative snapshot of the S protein and definition of angles/dihedrals measured to characterize the stalk motion. (B) Probability distribution of the bending angle for each HR2 linker and HR2-TM linker model. (C) Probability distributions of tilt angles for the resampled S protein structures compared to the experimental observation.^13^ The tilting angle is defined by the principal axis of the S protein head and the membrane normal.

We have performed 1.25 μs all-atom MD simulations of each of 16 models (i.e., a total of 20 μs) each containing about 2.3 million atoms (see Methods). Conformational analysis tools (CAT, http://www.md-simulations.de/CAT/) were used for high-throughput analysis of all simulation trajectories. The root-mean-square deviation (RMSD) time series in Figure S1 shows that the head region of the S protein (residue 1-1140) remains stable during the simulations with RMSD values of 4–5 Å in most systems. The stalk region, however, exhibits highly flexible motions at the HR2 linker and HR2-TM (see Movies S1–S2), which is consistent with S protein structures observed in cryo-ET.^13^ To further understand the flexible stalk motion, the bending characteristics of the two linker regions, defined as θ_1_ and θ_2_ (Figure 2A), were quantified. Figure 2B shows the distributions of θ_1_ and θ_2_ for each model. Both M1 and M2 of θ_1_ show similar angle distributions centered at 150° (±15°) and 155° (±12°), respectively. The HR2-TM region, however, exhibited different bending motions. The M1 of HR2-TM shows a narrow distribution centered at 172° (±4°), whereas the M2 of HR2-TM shows a wide distribution centered at 155° (±14°). Twisting motions were also dependent on the HR2-TM model (Figure S5A). While both M1 and M2 of the HR2 linker show similar twist angle distributions (ϕ) centered at 66° (±46°) and 68° (±49°), respectively, the M1 of HR2-TM shows a narrow distribution centered at 99° (±18°) and the M2 of HR2-TM shows a wide distribution centered at 98° (±71°).

These bending and twisting characteristics are consistent with the secondary structure analysis. The secondary structures of HR2 linker M1 and M2 models are mostly in coil conformations during the simulation although local folding and unfolding occur in both models (Figure S2). The secondary structure of the initial HR2-TM M1 model mainly consists of helical structures that are mostly retained during the simulation time. On the other hand, the secondary structure of M2 initially modeled with turns and bends shows low helicity in the range of L1200–K1215 (Figure S3). This indicates that the flexible motions of the HR2-TM linker are strongly influenced by the secondary structure and initial model (within the current simulation time). Although the secondary structures of CP domains are different in the two models, they have no significant effect on the motions of the stalk (Figure S4). To further characterize the bending motions, the Pearson correlation coefficient (r) was calculated for all combinations of the HR2 linker and HR2-TM models (Figure S5B). The r values of all cases range from −0.16 to 0.13, indicating that there is no correlation between the bending motions of the HR2 linker and HR2-TM; thus, each linker acts as an independent hinge.

Although 16 × 1.25 μs MD simulations were performed, they do not cover all possible configurations of the S protein, especially with such flexible two linkers. To increase sampling, utilizing the independent θ_1_ and θ_2_ characteristics, S protein orientation was resampled based on three regions: head-HR1, HR1-HR2, and HR2-TM. First, 30 HR2-TM conformations were randomly extracted from each trajectory (excluding HR2-TM M1 models), and their TM domain was superimposed to the TM domain of the initial model to resample HR2 domain motions. Second, 30 HR1-HR2 conformations were randomly extracted and superimposed to each of the resampled HR2 domains. Finally, 30 head-HR1 conformations were randomly extracted and superimposed to the resampled HR1 domains. In summary, 27 000 configurations of the S protein on a viral membrane were generated. Figure 2C shows the tilt angle of the resampled configurations of the S protein using M1 and M2 for the HR2 linker and only M2 for HR-TM. The S protein can tilt by up to 90° toward the membrane and tilt angles around 48° are most probable. This tilt angle distribution agrees well with the experimental observation.^11,13^ However, if M1 was used for HR2-TM, the tilt angle distribution of the S protein becomes narrow (Figure S6). This indicates that both M1 and M2 of the HR2 linker are reliable models, but for HR2-TM, M2 is more appropriate to represent S protein configurations. To further understand the contribution of each independent hinge motion on the tilt angle, the S protein was resampled separately with HR2-TM only and with HR1-HR2 only. In both cases, the resampled S protein shows a narrow-angle distribution compared to the experimental observation (Figure 2C), indicating that both linkers are necessary for the full tilting motions of the S protein observed in the experiment.

### 3.2 Flexibility of the Stalk May Facilitate S Protein Binding to ACE2

**Figure 3:**
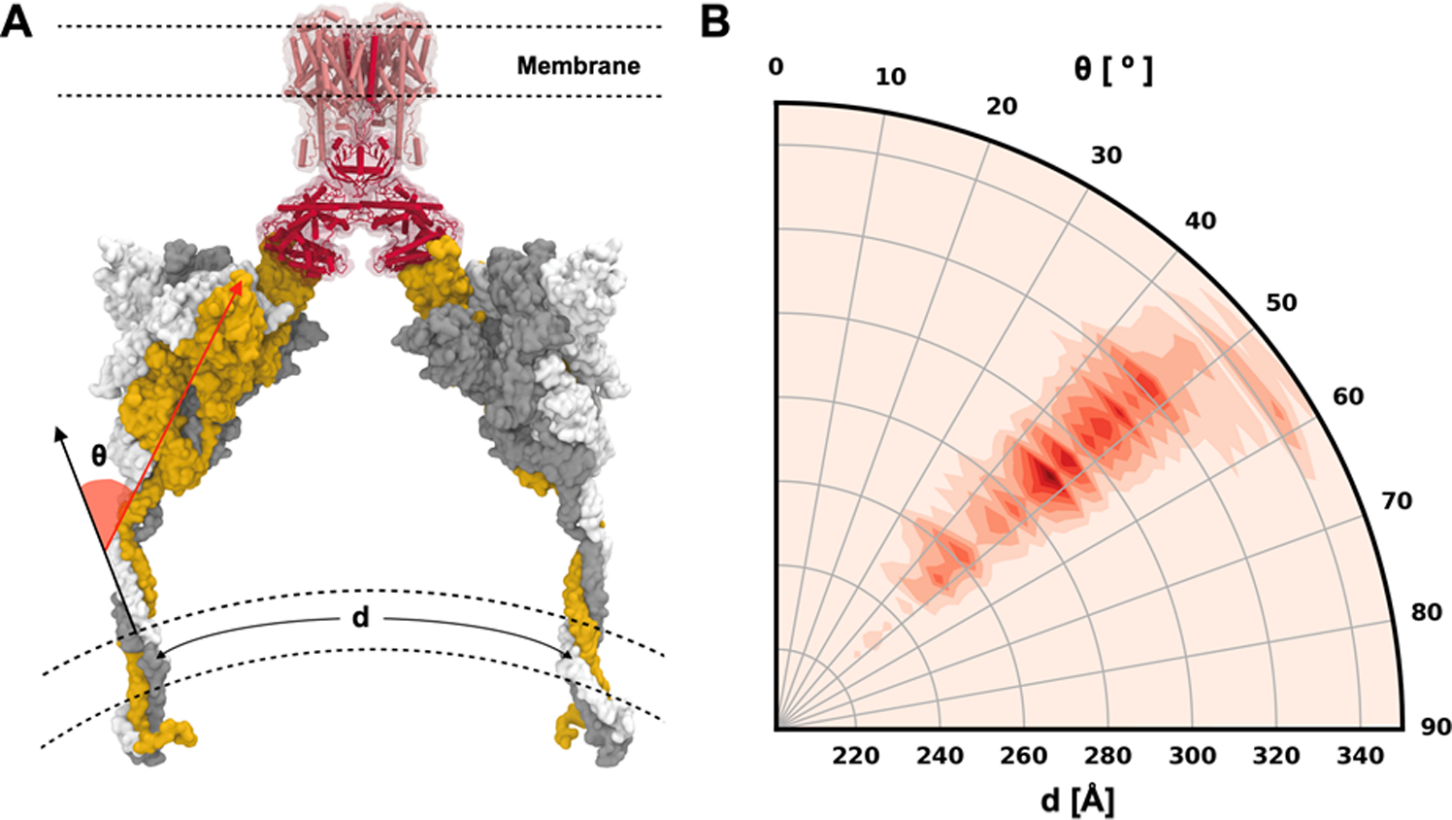
S protein configurations competent for ACE2 binding. (A) Illustrative snapshot of the S protein–ACE2-B^0^AT1 complex. Three individual chains of the S protein are colored in yellow, gray, and white, and ACE2 and B^0^AT1 are represented as red and pink, respectively. (B) Distribution of the tilting angle (θ) as a function of the arc length (*d*) between the centers of mass (COMs) of TM domains.

To explore the effect of flexible stalk motion on ACE2 binding, we performed the structural alignment of the S protein to ACE2. The RBD in the complex with full-length human ACE2 in the presence of the neutral amino acid transporter B^0^AT1 (PDB: 6M17^10^) was used for alignment. Fully independent bending and twisting motions of two stalk linkers allow us to increase the number of S protein samples. 125 head-HR1, HR1-HR2, and HR2-TM-CP conformations were separately extracted from each trajectory with a 10 ns interval. Each RBD of head-HR1 conformations was first superimposed to the RBD-ACE2-B^0^AT1 complex. Then, the HR1-HR2 conformations were superimposed to each of HR1 from the previous step. Finally, the HR2-TM-CP conformations were superimposed to each of HR2 from the previous step. Figure 3A shows one of the most probable configurations of the S protein–ACE2 complex. The tilting angle (θ) is defined in Figure 2, and the distance (*d*) is defined by an arc length between the centers of mass (COMs) of two TM domains. As shown in Figure 3B, *d* ranges from 240 to 350 Å and θ ranges from 30 to 60°. At the most probable configuration, *d* and θ are about 290 Å and 46°, respectively. Note that there is approximately one S protein per 1000 nm^2^ (316 Å × 316 Å) on the viral surface.^14^ This sparse distribution of the S protein suggests that receptor binding can be promoted by enough space to have orientational degrees of freedom for the RBD. Moreover, it is reported that the most probable tilting angle of the prefusion state is about 40–50°^11,13^ (also see Figure 2). This tilting angle appears to maximize the accessibility of the receptor-binding motif to ACE2 (when the RBD is in an open conformation), which could account for the high infection rate of SARS-CoV-2.

### 3.3 Glycans Influence RBD and NTD Motions and Contribute to S Trimer Stability

**Figure 4:**
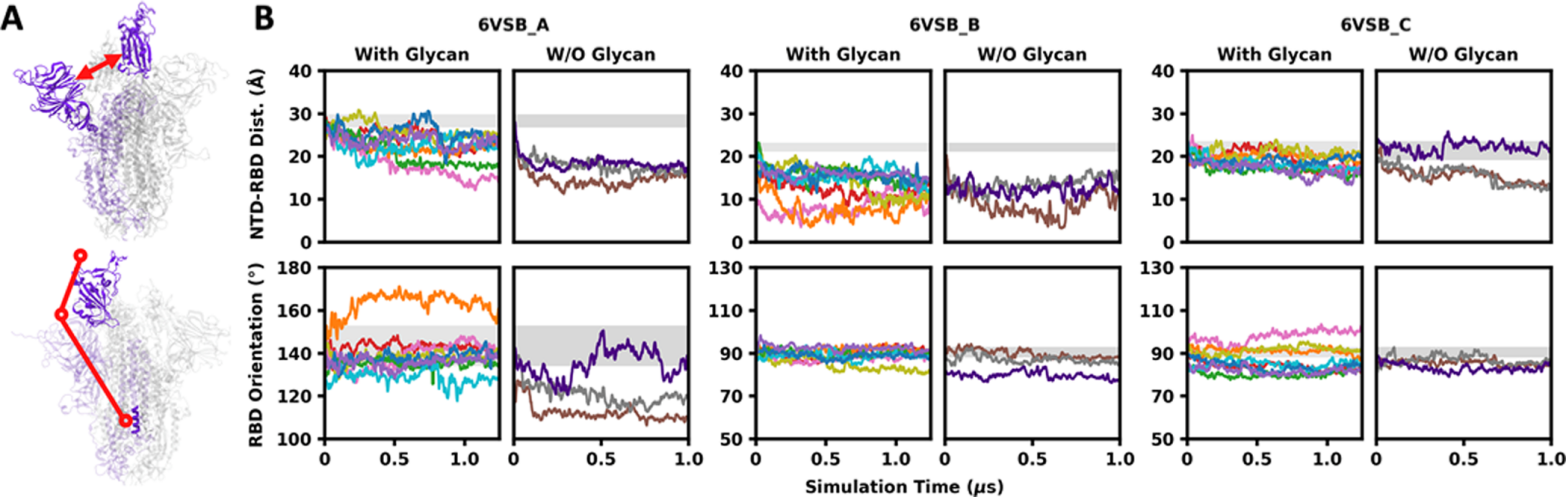
Motions of the RBD and the NTD in fully glycosylated systems and non-glycosylated systems. (A) Illustration of the NTD–RBD distance (*d*) and the RBD orientation angle (θ). (B) Time series of *d* and θ in three chains of 6VSB. *d* is defined by the minimum distance between the RBD (N334 to P527) and the NTD (C15 to S305). θ is defined by three points corresponding to the (i) COM of L452 and L492, (ii) COM of N334, and (iii) COM of S1030. The ranges of *d* and θ observed in the available PDB S protein structures are shaded by gray regions.

To explore RBD and NTD motions, we measured two structural features (Figure 4A): the RBD–NTD distance (*d*) defined by the minimum distance between RBD and NTD, and the RBD orientation angle (θ) defined by two points at each end of the RBD and the third point on the center axis of the S trimer. One RBD (in both open and closed states) forms a U-shaped pocket with the NTD in the same monomer, which is occupied by the neighboring RBD in a closed state. Therefore, *d* estimates the RBD–NTD pocket size and θ estimates the extent of RBD opening. The time series of *d* and θ in 6VSB and 6VXX are shown in Figure 4B and S7, respectively. In cryo-EM structures available in the PDB, θ in the open-state RBD ranges from 134 to 153°, and θ in the close-state RBD ranges from 88 to 93°. The trajectories of fully glycosylated models completely cover the RBD orientation angles observed in cryo-EM structures and explore a wider range of the conformational space. In particular, θ of 6VSB_A ranges from 120 to 170°, indicating that the open-state RBD is much more flexible than the closed-state RBD, and it is consistent with the RMSD and root-mean-square fluctuation (RMSF) results shown in Figure S1. For both 6VSB_A and 6VSB_C, the simulations cover the NTD–RBD distances observed in the PDB cryo-EM structures. In 6VSB_B, the pocket formed by NTD and RBD (chain B) is empty due to the opening of the neighboring RBD (chain A), and consequently, the NTD moved close to the RBD.

To investigate the impact of glycans on the transition between RBD open and closed states, we built and simulated non-glycosylated head-only systems (three replicates for 6VSB and 6VXX) by removing all N-/O-linked glycans and truncating the stalk. It is worth noting that the RBD in non-glycosylated 6VSB_A started to close at the very beginning of trajectories in all three replicas (Figure 4B). In the trajectory (colored in brown), θ decreases to 110°, which is about in the middle of open and closed states, and in the trajectory (colored in purple), the RBD reverted to opening at around 0.25 μs. Since the transition between RBD open and closed states is a complicated process involving the motions of multiple domains and attached glycans, it may require a much longer simulation time to capture the conformation in which the geometries of both the protein and glycan satisfy the requirement for the transition to occur. Nonetheless, dramatic transitions from RBD open to more closed states in non-glycosylated 6VSB_A indicate that the open-state RBD is unstable when the glycans are removed. This implies that glycosylation plays critical roles in the viral entry as the RBD needs to be open to interact with ACE2.

**Figure 5:**
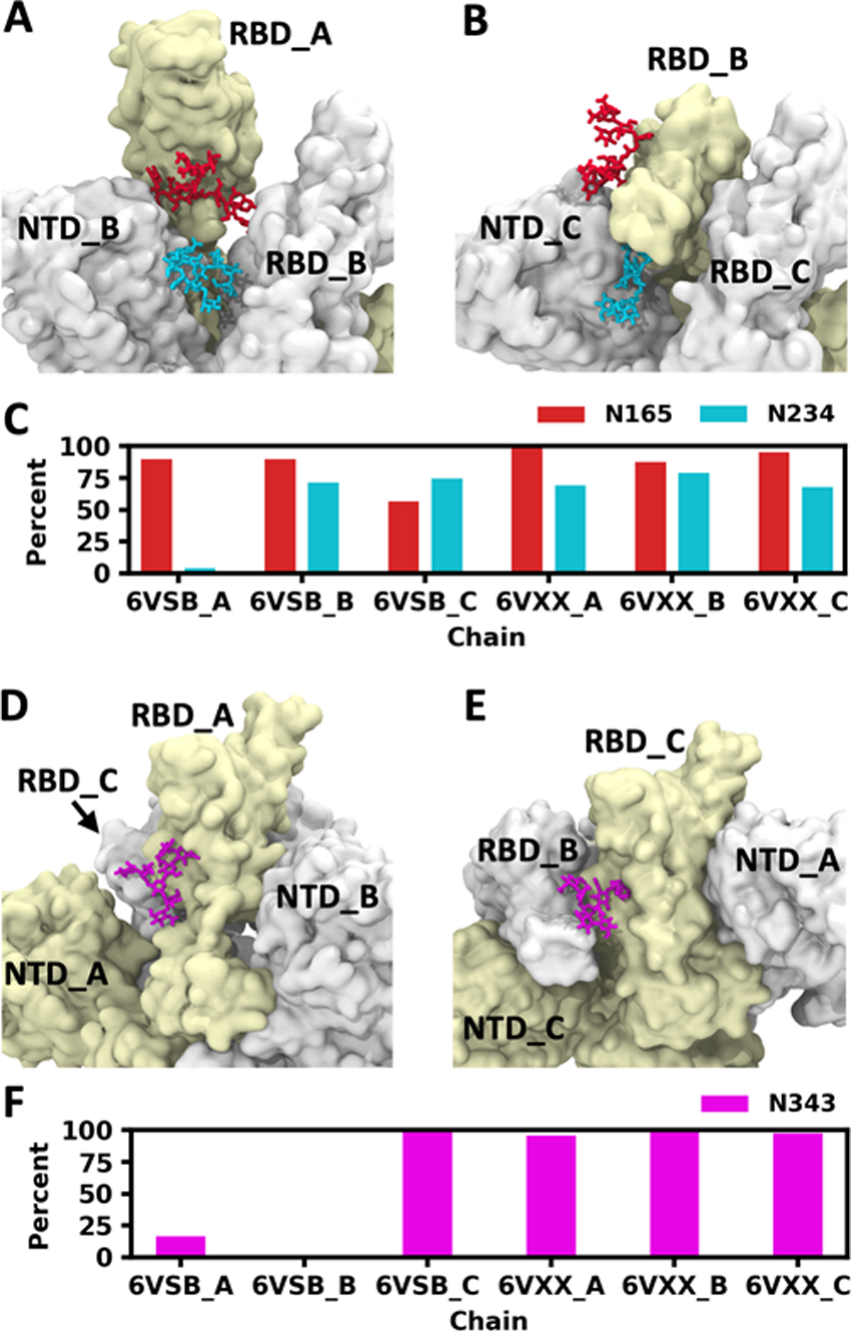
Critical roles of N165, N234, and N343 glycans in the transition between RBD open and closed conformations. (A) The open-state RBD (6VSB_A, pale yellow) is above N165 (red) and N234 (cyan) glycans on the neighboring NTD. (B) The closed-state RBD (6VSB_B) is between N165 and N234 glycans. (C) Frequency of contacts between the RBD and two glycans (N165 and N234). (D) N343 (magenta) glycan on the open-state RBD (6VSB_A) is free. (E) N343 glycan on the closed-state RBD (6VSB_C) interacts with the neighboring closed-state RBD (6VSB_C). (F) Frequency of contacts between N343 glycan and the neighboring RBD.

We identified three N-glycans that have important roles in the conformational change of the RBD. They are attached to N165 and N234 on the NTD and N343 on the RBD. When RBD is open (6VSB_A), N165 and N234 glycans on the NTD of the neighboring chain (6VSB_B) are both located below the open-state RBD (Figure 5A), which holds the open-state RBD. Although both are near the open-state RBD, only N165 glycan has frequent contacts with the RBD (>85% of snapshots) (Figure 5C), and N234 glycan interacts with the open-state RBD occasionally (<5% of snapshots), which is different from the findings from Casalino et al’s study.^16^ Such a difference could attribute to the initial models and simulation lengths. When the RBD is closed (all except 6VSB_A), the RBD forms a sandwich-like arrangement with two glycans. N165 glycan is located above the RBD and N234 glycan is located below the RBD (Figure 5B). Both glycans frequently interact with the closed-state RBD (Figure 5C), which makes the transition to an open conformation hard. The glycan attached to N343 on the RBD orients toward the solvent and hardly interacts with other domains when the RBD is open (6VSB_A) (Figure 5D). When the RBD is closed (6VSB_B) but the neighboring RBD (6VSB_A) is open, N343 glycan orients toward the pocket between the NTD and the RBD and interacts with N165 glycan, which makes the open to closed state transition of the neighboring RBD difficult. When the RBD is closed (all expect 6VSB_A and 6VSB_B) and the neighboring RBD is also closed, N343 glycan makes extensive interactions with the neighboring RBD (Figure 5E), which contributes to the stability of both RBDs in a closed conformation. The atom contacts between RBD N343 glycan and the neighboring RBD exist in more than 95% of snapshots when both RBDs are closed (Figure 5F). Given that the RBD is shown to constantly transit between open and closed states in the experiment, we propose that glycans serve as a clutch that temporarily holds the RBD in an open or closed conformation, which modulates the lifetime of both open and closed states.

In addition, we calculated the accessible surface area (ASA) reduced due to the formation of the S trimer. For example, the ASA reduction for chain A was calculated by S_A_ + S_BC_ – S_ABC_, where S_A_, S_BC_, and S_ABC_ are the ASA of chain A, chains BC-only complex, and chains ABC complex (i.e., S trimer), respectively. The ASA reduction due to trimer formation was split into the portion from protein only and the portion involving glycans. The trimer interface interaction involving glycans is about 30% when the entire S1 subunit is considered, and it increases to about 40% when only the RBD and the NTD are considered (Figure S8). This suggests that glycans make significant contributions to the stability of the S trimer, which is different from the common belief that protein–protein interactions are the only dominating factor to the stability of a protein multimer.

### 3.4 Impact of Glycan Shielding on Antibody Binding is Overestimated

**Figure 6:**
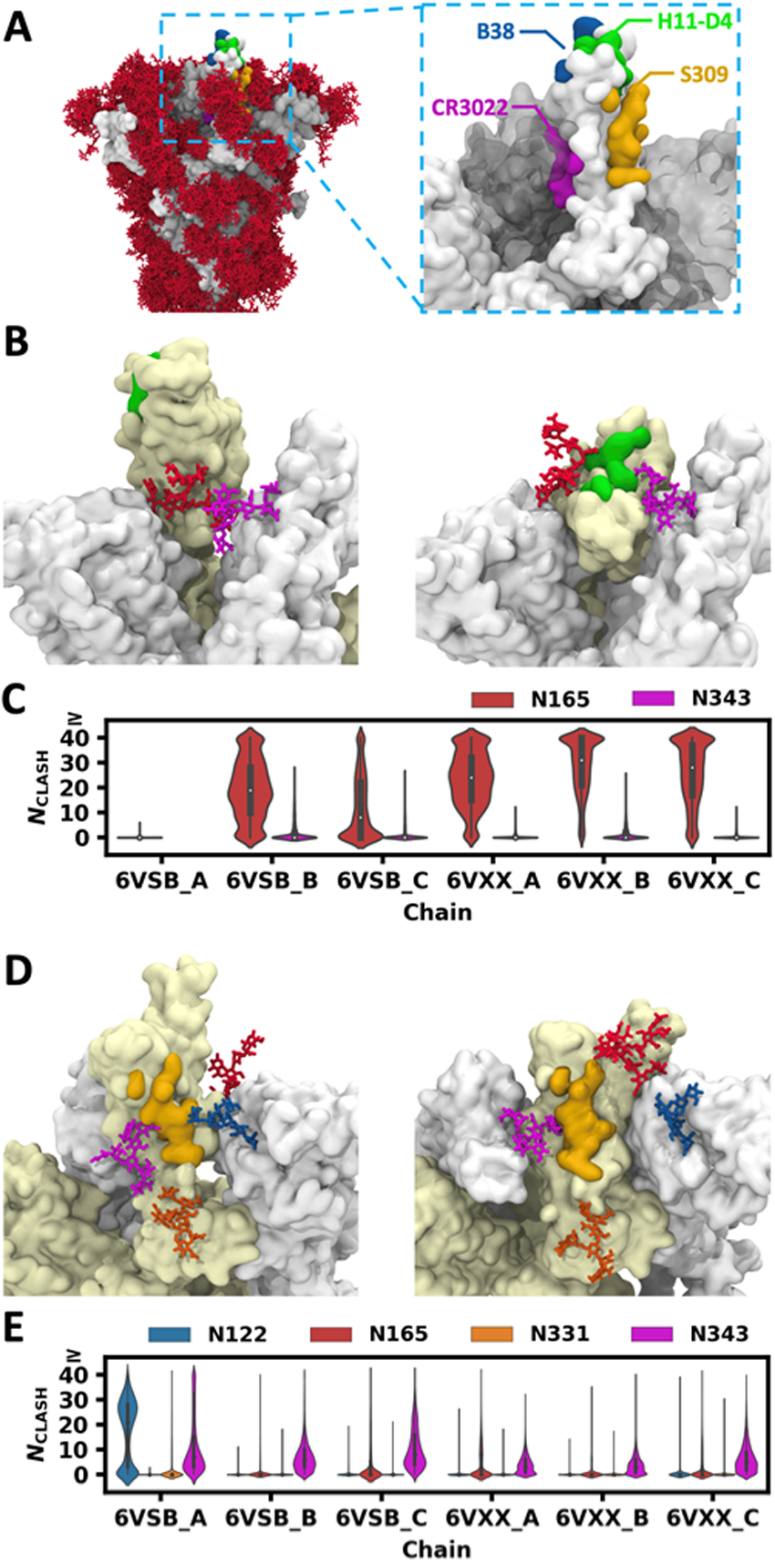
Clash between glycans and superimposed antibodies. (A) Distribution of glycans when S head structures in multiple snapshots are aligned (left). Four epitopes targeted by neutralizing antibodies are shown in different colors (right). (B) H11-D4 epitope (green) in the RBD (left: open, right: closed), N165 glycan (red) on the neighboring NTD, and N343 glycan (violet) on the neighboring RBD. (C) Distributions of glycan heavy atom numbers in clash (*N*_CLASH_) with the superposed H11-D4 antibody. (D) S309 epitope (yellow) and N331 (orange) and N343 (violet) glycans on the RBD (left: open, right: closed), N122 (blue) and N165 (red) glycans on the neighboring NTD. (E) Distributions of *N*_CLASH_ with the superimposed S309 antibody.

Viruses evolve to minimize the immunogenicity by coating the exposed viral proteins with non-immunogenic or weakly immunogenic glycans. It is commonly believed that the glycans on the viral envelope shield viruses from the host immune system. To get an impression of such glycan shielding, we aligned the S head in each trajectory snapshot and the glycan distributions are shown in Figure 6A. Most glycans are very flexible and they move around in a wide range of space, which covers most of the trimer surface. However, the role of glycan is not limited to shielding. During the past decade, many glycan-dependent HIV neutralizing antibodies have been discovered and extensively studied, which target both the envelope protein and glycans.^48−50^ In the cryo-EM structure of the S trimer in the complex with the S309 antibody (PDB ids: 6WPS and 6WPT(51)), S309 interacts with the glycan attached to N343.

To explore the role of glycans in antibody binding, we used TM-align^52^ to superimpose the RBD in RBD–antibody complex structures in the PDB onto the RBD in each simulation snapshot and calculated the number of glycan heavy atoms that have clashes with the antibodies (*N*_CLASH_, Figure S9). In this work, we discuss four RBD-targeting antibodies, namely, B38, CR3022, H11-D4, and S309 (Figure 6A). The epitopes of B38 and CR3022 are irrelevant to glycans. B38 binds to the same region of RBD as ACE2 does. This epitope is fully exposed in the open-state RBD, but when the RBD is closed, the B38 epitope is masked by the neighboring RBD in either the open or closed state (Figure S10A). The epitope of CR3022 is on the inner surface of the RBD, and it is only accessible when all three RBD are open (Figure S10B).

The epitope of H11-D4 is next to the epitope of B38, and it is also fully exposed when the RBD is open. When the RBD is closed, the glycans attached to N165 and N343 on the neighboring chain are located near this epitope (Figure 6B and S10C). As shown in the distribution of *N*_CLASH_ with H11-D4 (Figure 6C), N343 glycan rarely interferes with the antibody, but N165 glycan has high probabilities to make severe clashes with the antibody when the RBD is closed. For comparison, we also aligned a nanobody to the RBD in each simulation snapshot. Though N165 glycan still frequently clashes with the nanobody, the frequency of severe clashes is much lower (Figure S11); thus, there is a chance that a nanobody can bind to this epitope as shown in PDB id 6Z43.

The epitope of S309 is surrounded by four glycans. Two of them are attached to N331 and N343 on the same RBD, and the other two are attached to N122 and N165 on the neighboring NTD (Figure 6D and S10D). The distributions of *N*_CLASH_ with S309 are shown in Figure 6E. N165 and N331 glycans rarely interfere with the S309 antibody in both open- and closed-state RBD. N343 glycan has minor clashes with the antibody in most snapshots, and such minor clashes are not sufficient to block antibody binding, as these clashes can be easily removed with small changes in glycan conformation and orientation. The antibody–glycan interactions can also contribute to antibody binding, which is observed in the cryo-EM structures. In more than half of all snapshots, N122 glycan has severe clashes with the antibody when the RBD is open, but it moves away from the superposition of the antibody in the remaining snapshots. This suggests that the S309 epitope in the open-state RBD is blocked by N122 glycan in more than half of the simulation time, but it is still accessible to the antibody.

As a comparison, we calculated the ASA of S309 and H11-D4 epitopes using a probe radius of 7.2 Å that is commonly used to approximate the size of hypervariable loops of the antibody and compared the epitope ASA with and without glycans (Figure S12). For H11-D4, we observed significant decreases in the epitope ASA in all chains except 6VSB_A and 6VSB_C, which is generally consistent with the frequency of clashes between glycans and antibodies. However, for S309, the epitope ASA decreases significantly in all chains when glycans are present. This is contradictory to the result that N343 glycans occasionally have only slight clashes with the superimposed antibody when the RBD is closed (all except 6VSB_A). In addition, the PDB structures of the S trimer in the complex with S309 show that N343 glycan interacts with the antibody. In the calculation of ASA, a point on the surface is considered inaccessible even if the probe sphere has a very tiny clash with the molecule. However, the shape of the antibody is not a sphere and it can have narrow-shaped regions that extend deeply into the pocket in the epitope. A glycan like the one attached to N343 can reduce the epitope ASA even though it may contribute to antibody binding. Therefore, in some cases, a simple comparison of protein ASA with and without glycans is likely to overestimate the impact of glycan shielding.

## 4 Conclusions

In this work, we present multiple μs-long all-atom MD simulations of the fully glycosylated full-length S protein in a viral membrane. Our MD simulations reveal the overall shape of the S protein, and its orientation on the membrane surface is determined by a highly flexible stalk composed of two independent joints. Importantly, S protein models from our simulations allow us to predict the possible configurations of the S protein–ACE2-B^0^AT1 complex with allowable orientations and distances between two S proteins on the membrane surface. The simulation here also provides insights into how glycans influence the open/closed state change of the RBD and the antibody binding to RBD epitopes. We identify glycans attached to multiple glycosylation sites that stabilize the open and/or closed states of the RBD by making a high energetic barrier between the open–closed transition. The simulation of non-glycosylated systems shows that the open-state RBD becomes unstable when glycans are removed and the transition to the closed state occurred at the early stage of the simulation. By aligning the RBD–antibody complex structures to the simulation trajectories, we reveal that the impact of glycan shielding is overestimated by a simple ASA analysis. More importantly, glycans not only serve as shields for immune evasion but also contributes to antibody binding. Our work sheds light on the full structure and dynamics of the S protein and we hope our work to be useful for the development of vaccines and antiviral drugs.

## Supporting Information Available

RMSF and RMSD of the S protein head (Figure S1); secondary structure analysis of HR2 linker (A) model 1 and (B) model 2 (Figure S2); secondary structure analysis of HR2-TM linker (A) model 1 and (B) model 2 (Figure S3); secondary structure analysis of CP domain (A) model 1 and (B) model 2 (Figure S4); twist and bending motions of the S protein in a viral membrane (Figure S5); tilt angle of the S protein in a viral membrane (Figure S6); motions of the RBD and the NTD in fully glycosylated systems and non-glycosylated systems (6VXX) (Figure S7); surface area buried in the interface of the S trimer (Figure S8); procedure of measuring the number of glycan atoms clashing with the superimposed antibody (Figure S9); four antibodies are aligned onto their epitopes in the S trimer (Figure S10); clash between glycans and the superimposed H11-D4 nanobody (Figure S11); and accessible surface areas of antibody epitopes when glycans are present or removed (Figure S12)
(PDF)
Highly flexible motions at the HR2 linker and HR2-TM of 6VSB_1_1_1 system (Movie 1) (MP4)
Highly flexible motions at the HR2 linker and HR2-TM of 6VXX_2_2_2 system (Movie 2) (MP4)

## References

[ref1] Lai M. M.; Cavanagh D. The molecular biology of coronaviruses. Adv. Virus Res. 1997, 48, 1–100. 10.1016/s0065-3527(08)60286-9. 9233431 PMC7130985

[ref2] Letko M.; Marzi A.; Munster V. Functional assessment of cell entry and receptor usage for SARS-CoV-2 and other lineage B betacoronaviruses. Nat. Microbiol. 2020, 5, 562–569. 10.1038/s41564-020-0688-y. 32094589 PMC7095430

[ref3] Hoffmann M.; Kleine-Weber H.; Schroeder S.; Krüger N.; Herrler T.; Erichsen S.; Schiergens T. S.; Herrler G.; Wu N.-H.; Nitsche A.; et al. SARS-CoV-2 cell entry depends on ACE2 and TMPRSS2 and is blocked by a clinically proven protease inhibitor. Cell 2020, 181, 271–280. 10.1016/j.cell.2020.02.052. 32142651 PMC7102627

[ref4] Wrapp D.; Wang N.; Corbett K. S.; Goldsmith J. A.; Hsieh C.-L.; Abiona O.; Graham B. S.; McLellan J. S. Cryo-EM structure of the 2019-nCoV spike in the prefusion conformation. Science 2020, 367, 1260–1263. 10.1126/science.abb2507. 32075877 PMC7164637

[ref5] Cao Y.; Su B.; Guo X.; Sun W.; Deng Y.; Bao L.; Zhu Q.; Zhang X.; Zheng Y.; Geng C.; et al. Potent neutralizing antibodies against SARS-CoV-2 identified by high-throughput single-cell sequencing of convalescent patients’ B cells. Cell 2020, 182, 73–84. 10.1016/j.cell.2020.05.025. 32425270 PMC7231725

[ref6] Yuan M.; Wu N. C.; Zhu X.; Lee C.-C. D.; So R. T.; Lv H.; Mok C. K.; Wilson I. A. A highly conserved cryptic epitope in the receptor binding domains of SARS-CoV-2 and SARS-CoV. Science 2020, 368, 630–633. 10.1126/science.abb7269. 32245784 PMC7164391

[ref7] Shi R.; Shan C.; Duan X.; Chen Z.; Liu P.; Song J.; Song T.; Bi X.; Han C.; Wu L.; et al. A human neutralizing antibody targets the receptor binding site of SARS-CoV-2. Nature 2020, 584, 120–124. 10.1038/s41586-020-2381-y. 32454512

[ref8] Ju B.; Zhang Q.; Ge J.; Wang R.; Sun J.; Ge X.; Yu J.; Shan S.; Zhou B.; Song S.; et al. Human neutralizing antibodies elicited by SARS-CoV-2 infection. Nature 2020, 584, 115–119. 10.1038/s41586-020-2380-z. 32454513

[ref9] Hoffmann M.; Kleine-Weber H.; Pöhlmann S. A multibasic cleavage site in the spike protein of SARS-CoV-2 is essential for infection of human lung cells. Mol. Cell 2020, 78, 779–784. 10.1016/j.molcel.2020.04.022. 32362314 PMC7194065

[ref10] Yan R.; Zhang Y.; Li Y.; Xia L.; Guo Y.; Zhou Q. Structural basis for the recognition of SARS-CoV-2 by full-length human ACE2. Science 2020, 367, 1444–1448. 10.1126/science.abb2762. 32132184 PMC7164635

[ref11] Yao H.; Song Y.; Chen Y.; Wu N.; Xu J.; Sun C.; Zhang J.; Weng T.; Zhang Z.; Wu Z. Molecular architecture of the SARS-CoV-2 virus. Cell 2020, 183, 1–9. 10.1016/j.cell.2020.09.018. 32979942 PMC7474903

[ref12] Walls A. C.; Park Y.-J.; Tortorici M. A.; Wall A.; McGuire A. T.; Veesler D. Structure, function, and antigenicity of the SARS-CoV-2 spike glycoprotein. Cell 2020, 181, 281–292. 10.1016/j.cell.2020.02.058. 32155444 PMC7102599

[ref13] Turoňová B.; Sikora M.; Schürmann C.; Hagen W. J.; Welsch S.; Blanc F. E.; von Bülow S.; Gecht M.; Bagola K.; Hörner C.; et al. In situ structural analysis of SARS-CoV-2 spike reveals flexibility mediated by three hinges. Science 2020, 370, 203–208. 10.1126/science.abd5223. 32817270 PMC7665311

[ref14] Ke Z.; Oton J.; Qu K.; Cortese M.; Zila V.; McKeane L.; Nakane T.; Zivanov J.; Neufeldt C. J.; Cerikan B.; et al. Structures and distributions of SARS-CoV-2 spike proteins on intact virions. Nature 2020, 588, 498–502. 10.1038/s41586-020-2665-2. 32805734 PMC7116492

[ref15] Grant O. C.; Montgomery D.; Ito K.; Woods R. J. Analysis of the SARS-CoV-2 spike protein glycan shield: implications for immune recognition. Sci. Rep. 2020, 10, 14991 10.1038/s41598-020-71748-7. 32929138 PMC7490396

[ref16] Casalino L.; Gaieb Z.; Goldsmith J. A.; Hjorth C. K.; Dommer A. C.; Harbison A. M.; Fogarty C. A.; Barros E. P.; Taylor B. C.; McLellan J. S.; et al. Beyond Shielding: The Roles of Glycans in the SARS-CoV-2 Spike Protein. ACS Cent. Sci. 2020, 6, 1722–1734. 10.1021/acscentsci.0c01056. 33140034 PMC7523240

[ref17] Ko J.; Park H.; Seok C. GalaxyTBM: template-based modeling by building a reliable core and refining unreliable local regions. BMC Bioinf. 2012, 13, 198 10.1186/1471-2105-13-198. PMC3462707 22883815

[ref18] Ko J.; Lee D.; Park H.; Coutsias E. A.; Lee J.; Seok C. The FALC-Loop web server for protein loop modeling. Nucleic Acids Res. 2011, 39, W210–W214. 10.1093/nar/gkr352. 21576220 PMC3125760

[ref19] Park T.; Baek M.; Lee H.; Seok C. GalaxyTongDock: Symmetric and asymmetric ab initio protein–protein docking web server with improved energy parameters. J. Comput. Chem. 2019, 40, 2413–2417. 10.1002/jcc.25874. 31173387

[ref20] Croll T. I. ISOLDE: a physically realistic environment for model building into low-resolution electron-density maps. Acta Crystallogr., Sect. D: Struct. Biol. 2018, 74, 519–530. 10.1107/S2059798318002425. 29872003 PMC6096486

[ref21] Jo S.; Song K. C.; Desaire H.; MacKerell A. D. Jr; Im W. Glycan Reader: automated sugar identification and simulation preparation for carbohydrates and glycoproteins. J. Comput. Chem. 2011, 32, 3135–3141. 10.1002/jcc.21886. 21815173 PMC3188666

[ref22] Park S.-J.; Lee J.; Patel D. S.; Ma H.; Lee H. S.; Jo S.; Im W. Glycan Reader is improved to recognize most sugar types and chemical modifications in the Protein Data Bank. Bioinformatics 2017, 33, 3051–3057. 10.1093/bioinformatics/btx358. 28582506 PMC5870669

[ref23] Park S.-J.; Lee J.; Qi Y.; Kern N. R.; Lee H. S.; Jo S.; Joung I.; Joo K.; Lee J.; Im W. CHARMM-GUI Glycan Modeler for modeling and simulation of carbohydrates and glycoconjugates. Glycobiology 2019, 29, 320–331. 10.1093/glycob/cwz003. 30689864 PMC6422236

[ref24] Jo S.; Kim T.; Im W. Automated builder and database of protein/membrane complexes for molecular dynamics simulations. PLoS One 2007, 2, e880 10.1371/journal.pone.0000880. 17849009 PMC1963319

[ref25] Jo S.; Lim J. B.; Klauda J. B.; Im W. CHARMM-GUI Membrane Builder for mixed bilayers and its application to yeast membranes. Biophys. J. 2009, 97, 50–58. 10.1016/j.bpj.2009.04.013. 19580743 PMC2711372

[ref26] Wu E. L.; Cheng X.; Jo S.; Rui H.; Song K. C.; Dávila-Contreras E. M.; Qi Y.; Lee J.; Monje-Galvan V.; Venable R. M.; et al. CHARMM-GUI membrane builder toward realistic biological membrane simulations. J. Comput. Chem. 2014, 35, 1997–2004. 10.1002/jcc.23702. 25130509 PMC4165794

[ref27] Watanabe Y.; Allen J. D.; Wrapp D.; McLellan J. S.; Crispin M. Site-specific glycan analysis of the SARS-CoV-2 spike. Science 2020, 369, eabb9983 10.1126/science.abb9983. PMC7199903 32366695

[ref28] Shajahan A.; Supekar N. T.; Gleinich A. S.; Azadi P. Deducing the N-and O-glycosylation profile of the spike protein of novel coronavirus SARS-CoV-2. Glycobiology 2020, 30, 981–988. 10.1093/glycob/cwaa042. 32363391 PMC7239183

[ref29] Woo H.; Park S.-J.; Choi Y. K.; Park T.; Tanveer M.; Cao Y.; Kern N. R.; Lee J.; Yeom M. S.; Croll T.; et al. Modeling and Simulation of a Fully-glycosylated Full-length SARS-CoV-2 Spike Protein in a Viral Membrane. J. Phys. Chem. B 2020, 124, 7128–7137. 10.1021/acs.jpcb.0c04553. 32559081 PMC7341691

[ref30] Humphrey W.; Dalke A.; Schulten K. VMD: visual molecular dynamics. J. Mol. Graphics 1996, 14, 33–38. 10.1016/0263-7855(96)00018-5. 8744570

[ref31] Huang J.; Rauscher S.; Nawrocki G.; Ran T.; Feig M.; de Groot B. L.; Grubmüller H.; MacKerell A. D. CHARMM36m: an improved force field for folded and intrinsically disordered proteins. Nat. Methods 2017, 14, 71–73. 10.1038/nmeth.4067. 27819658 PMC5199616

[ref32] Klauda J. B.; Venable R. M.; Freites J. A.; O’Connor J. W.; Tobias D. J.; Mondragon-Ramirez C.; Vorobyov I.; MacKerell A. D. Jr; Pastor R. W. Update of the CHARMM all-atom additive force field for lipids: validation on six lipid types. J. Phys. Chem. B 2010, 114, 7830–7843. 10.1021/jp101759q. 20496934 PMC2922408

[ref33] Klauda J. B.; Monje V.; Kim T.; Im W. Improving the CHARMM force field for polyunsaturated fatty acid chains. J. Phys. Chem. B 2012, 116, 9424–9431. 10.1021/jp304056p. 22697583

[ref34] Guvench O.; Greene S. N.; Kamath G.; Brady J. W.; Venable R. M.; Pastor R. W.; Mackerell A. D. Jr Additive empirical force field for hexopyranose monosaccharides. J. Comput. Chem. 2008, 29, 2543–2564. 10.1002/jcc.21004. 18470966 PMC2882059

[ref35] Guvench O.; Hatcher E.; Venable R. M.; Pastor R. W.; MacKerell A. D. Jr CHARMM additive all-atom force field for glycosidic linkages between hexopyranoses. J. Chem. Theory Comput. 2009, 5, 2353–2370. 10.1021/ct900242e. 20161005 PMC2757763

[ref36] Hatcher E.; Guvench O.; MacKerell A. D. Jr CHARMM additive all-atom force field for aldopentofuranoses, methyl-aldopentofuranosides, and fructofuranose. J. Phys. Chem. B 2009, 113, 12466–12476. 10.1021/jp905496e. 19694450 PMC2741538

[ref37] Jorgensen W. L.; Chandrasekhar J.; Madura J. D.; Impey R. W.; Klein M. L. Comparison of simple potential functions for simulating liquid water. J. Chem. Phys. 1983, 79, 926–935. 10.1063/1.445869.

[ref38] Steinbach P. J.; Brooks B. R. New spherical-cutoff methods for long-range forces in macromolecular simulation. J. Comput. Chem. 1994, 15, 667–683. 10.1002/jcc.540150702.

[ref39] Essmann U.; Perera L.; Berkowitz M. L.; Darden T.; Lee H.; Pedersen L. G. A smooth particle mesh Ewald method. J. Chem. Phys. 1995, 103, 8577–8593. 10.1063/1.470117.

[ref40] Lee J.; Cheng X.; Swails J. M.; Yeom M. S.; Eastman P. K.; Lemkul J. A.; Wei S.; Buckner J.; Jeong J. C.; Qi Y.; et al. CHARMM-GUI input generator for NAMD, GROMACS, AMBER, OpenMM, and CHARMM/OpenMM simulations using the CHARMM36 additive force field. J. Chem. Theory Comput. 2016, 12, 405–413. 10.1021/acs.jctc.5b00935. 26631602 PMC4712441

[ref41] Van Der Spoel D.; Lindahl E.; Hess B.; Groenhof G.; Mark A. E.; Berendsen H. J. GROMACS: fast, flexible, and free. J. Comput. Chem. 2005, 26, 1701–1718. 10.1002/jcc.20291. 16211538

[ref42] Hess B.; Bekker H.; Berendsen H. J.; Fraaije J. G. LINCS: a linear constraint solver for molecular simulations. J. Comput. Chem. 1997, 18, 1463–1472. 10.1002/(SICI)1096-987X(199709)18:12<1463::AID-JCC4>3.0.CO;2-H.

[ref43] Nosé S. A molecular dynamics method for simulations in the canonical ensemble. Mol. Phys. 1984, 52, 255–268. 10.1080/00268978400101201.

[ref44] Hoover W. G. Canonical dynamics: Equilibrium phase-space distributions. Phys. Rev. A 1985, 31, 1695 10.1103/PhysRevA.31.1695. 9895674

[ref45] Parrinello M.; Rahman A. Polymorphic transitions in single crystals: A new molecular dynamics method. J. Appl. Phys. 1981, 52, 7182–7190. 10.1063/1.328693.

[ref46] Nosé S.; Klein M. Constant pressure molecular dynamics for molecular systems. Mol. Phys. 1983, 50, 1055–1076. 10.1080/00268978300102851.

[ref47] Hopkins C. W.; Le Grand S.; Walker R. C.; Roitberg A. E. Long-time-step molecular dynamics through hydrogen mass repartitioning. J. Chem. Theory Comput. 2015, 11, 1864–1874. 10.1021/ct5010406. 26574392

[ref48] McLellan J. S.; Pancera M.; Carrico C.; Gorman J.; Julien J.-P.; Khayat R.; Louder R.; Pejchal R.; Sastry M.; Dai K.; et al. Structure of HIV-1 gp120 V1/V2 domain with broadly neutralizing antibody PG9. Nature 2011, 480, 336–343. 10.1038/nature10696. 22113616 PMC3406929

[ref49] Walker L. M.; Huber M.; Doores K. J.; Falkowska E.; Pejchal R.; Julien J.-P.; Wang S.-K.; Ramos A.; Chan-Hui P.-Y.; Moyle M.; et al. Broad neutralization coverage of HIV by multiple highly potent antibodies. Nature 2011, 477, 466–470. 10.1038/nature10373. 21849977 PMC3393110

[ref50] Walker L. M.; Phogat S. K.; Chan-Hui P.-Y.; Wagner D.; Phung P.; Goss J. L.; Wrin T.; Simek M. D.; Fling S.; Mitcham J. L.; et al. Broad and potent neutralizing antibodies from an African donor reveal a new HIV-1 vaccine target. Science 2009, 326, 285–289. 10.1126/science.1178746. 19729618 PMC3335270

[ref51] Pinto D.; Park Y.-J.; Beltramello M.; Walls A. C.; Tortorici M. A.; Bianchi S.; Jaconi S.; Culap K.; Zatta F.; De Marco A.; et al. Cross-neutralization of SARS-CoV-2 by a human monoclonal SARS-CoV antibody. Nature 2020, 583, 290–295. 10.1038/s41586-020-2349-y. 32422645

[ref52] Zhang Y.; Skolnick J. TM-align: a protein structure alignment algorithm based on the TM-score. Nucleic Acids Res. 2005, 33, 2302–2309. 10.1093/nar/gki524. 15849316 PMC1084323

