# Supplementary material for: Structure, Dynamics, Receptor Binding, and Antibody Binding of the Fully Glycosylated Full-Length SARS-CoV-2 Spike Protein in a Viral Membrane
Source: J Chem Theory Comput. 2021 Mar 10;17(4):2479–87. doi: 10.1021/acs.jctc.0c01144 (PMC8047829; doi:10.1021/acs.jctc.0c01144)
Supplement: Supplementary file 1 — ct0c01144_si_001.pdf [file ct0c01144_si_001.pdf]

Supporting Information

Structure, Dynamics, Receptor Binding, and Antibody Binding of Fully-glycosylated Full-length SARS-CoV-2 Spike Protein in a Viral Membrane

Yeol Kyo Choi[1†], Yiwei Cao[1†], Martin Frank[2†], Hyeonuk Woo[3], Sang-Jun Park[1], Min Sun Yeom[4], Tristan I. Croll[5], Chaok Seok[3], and Wonpil Im[1*]

[†]These authors contributed equally to this study.

[1]Departments of Biological Sciences, Chemistry, Bioengineering, and Computer Science and Engineering, Lehigh University, Bethlehem, Pennsylvania 18015, USA

[2]Biognos AB, Box 8963, 40274 Göteborg, Sweden

[3]Department of Chemistry, Seoul National University, Seoul 08826, Republic of Korea

[4]Korean Institute of Science and Technology Information, Daejeon 34141, Republic of Korea

[5]Department of Haematology, Cambridge Institute for Medical Research, University of Cambridge, Cambridge CB2 0XY, UK

*Corresponding authors: wonpil@lehigh.edu

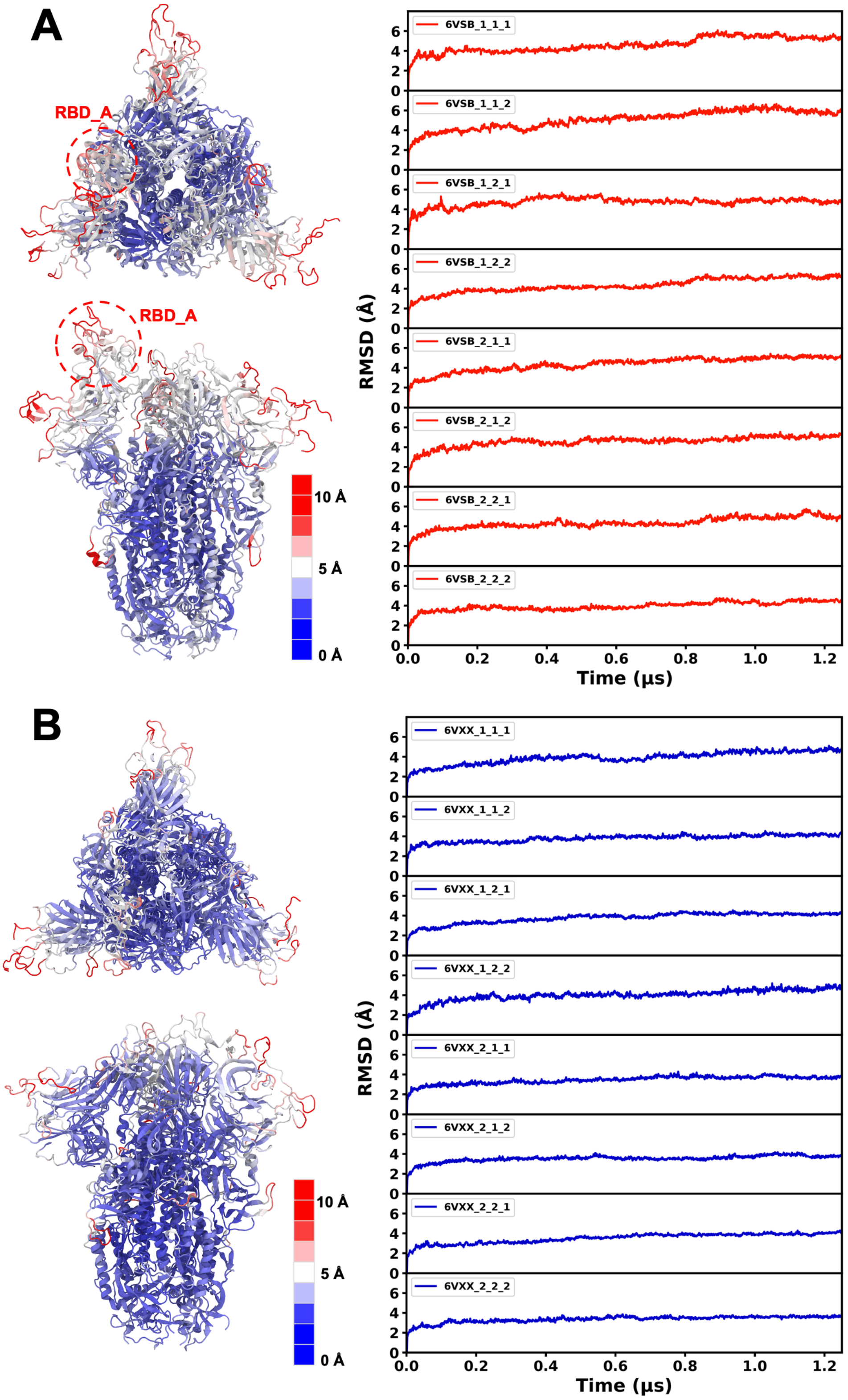

Figure S1. RMSF and RMSD of S protein head. (left) Root-mean-square-fluctuation (RMSF) and (right) time series of root-mean-square-deviation (RMSD) of S protein head (residue 1-1140) for (A) RDB up and (B) down conformations of chain A (RBD_A).

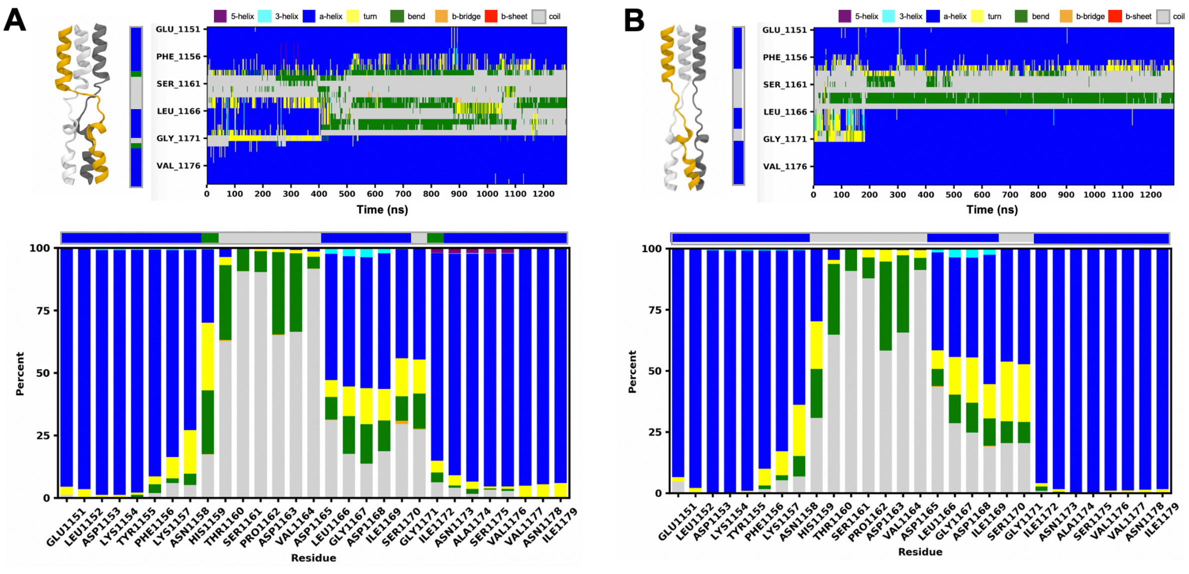

Figure S2. Secondary structure analysis of HR2 linker (A) model 1 and (B) model 2. (Top from right) a snapshot of model structure, an initial secondary structure, and time series of secondary structures of a representative system, and (bottom) secondary structure contents per residue from all simulations.

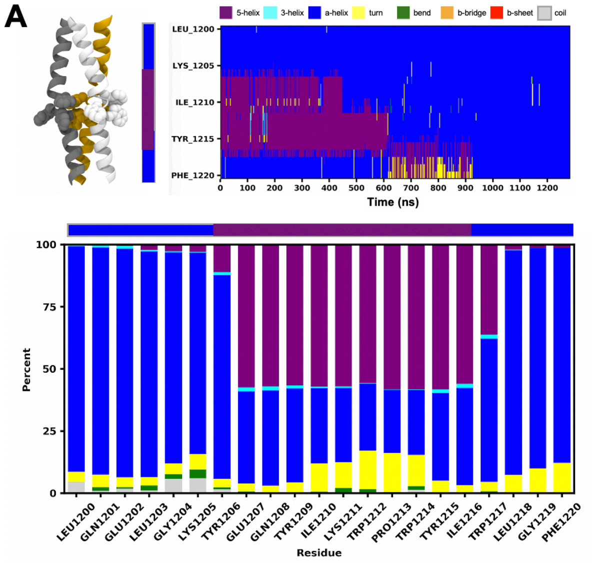

Figure S3. Secondary structure analysis of HR2-TM linker (A) model 1 and (B) model 2. (Top from right) a snapshot of model structure, an initial secondary structure, and time series of secondary structures of a representative system, and (bottom) secondary structure contents per residue from all simulations.

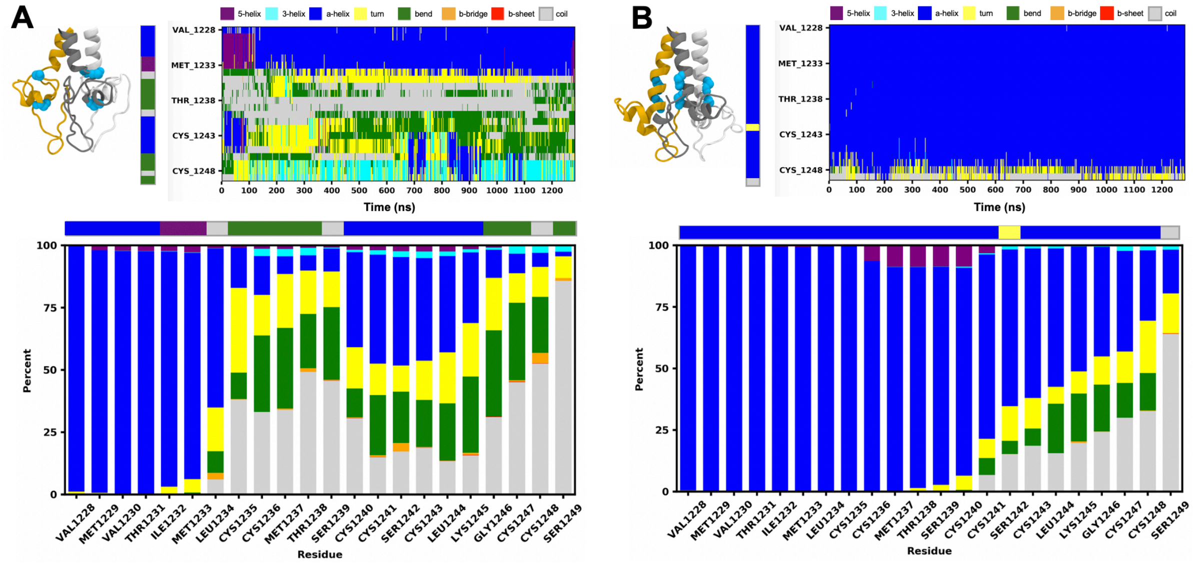

Figure S4. Secondary structure analysis of CP domain (A) model 1 and (B) model 2. (Top from right) a snapshot of model structure, an initial secondary structure, and time series of secondary structures of a representative system, and (bottom) secondary structure contents per residue from all simulations.

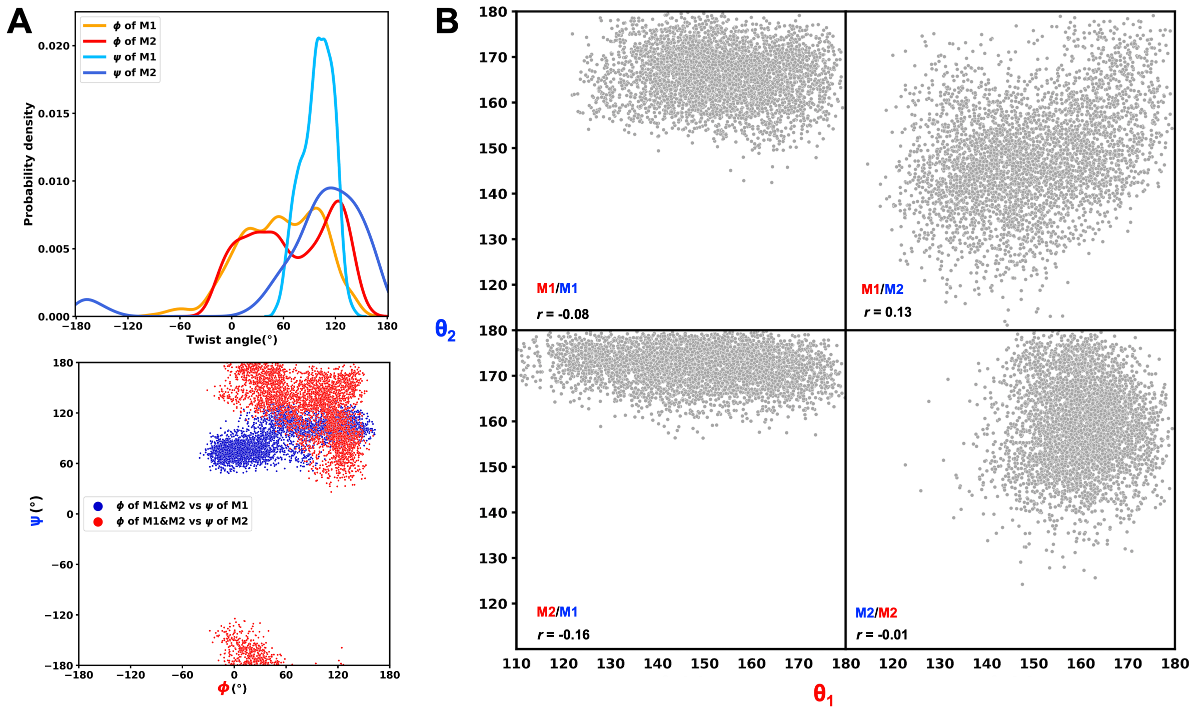

Figure S5. Twist and bending motions of S protein in a viral membrane. (A) (top) Probability distribution of twist angle for each HR2 linker and HR2-TM linker model and (bottom) scatter plots of twist angles of HR2 linker and HR2-TM linker. For HR2 linker, both M1 and M2 were used for analysis. (B) Scatter plots of bending angles for HR2 linker and HR2-TM linker models.

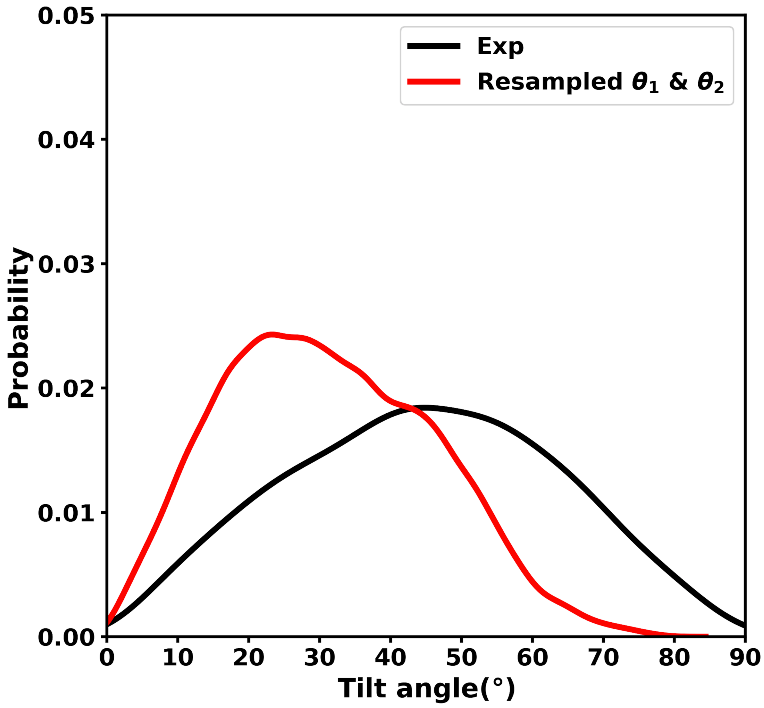

Figure S6. Tilt angle of S protein in a viral membrane. Probability distributions of tilt angles for the resampled S protein structures compared to the experimental observation. S protein configurations were resampled based on both M1 and M2 for HR1-HR2 region (θ_1) and only M2 for HR2-TM region (θ_2).

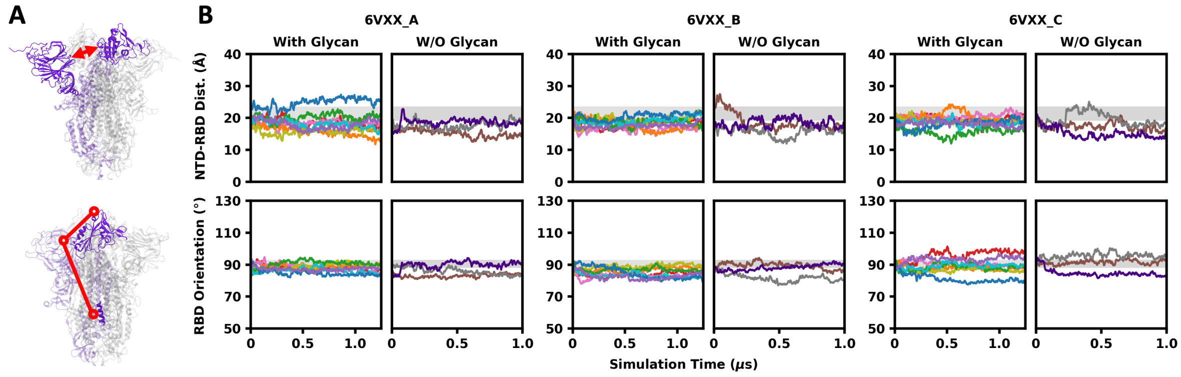

Figure S7. Motions of RBD and NTD in fully-glycosylated systems and non-glycosylated systems (6VXX). (A) Illustration of NTD-RBD distance (*d*) and RBD orientation angle (*θ*). (B) The time series of *d* and *θ* in three chains of 6VSB. *d* is defined by the minimum distance between RBD (N334 to P527) and NTD (C15 to S305). *θ* is defined by three points corresponding to the (i) COM of L452 and L492, (ii) COM of N334, and (iii) COM of S1030. The ranges of *d* and *θ* observed in available PDB S protein structures are shaded by gray regions.

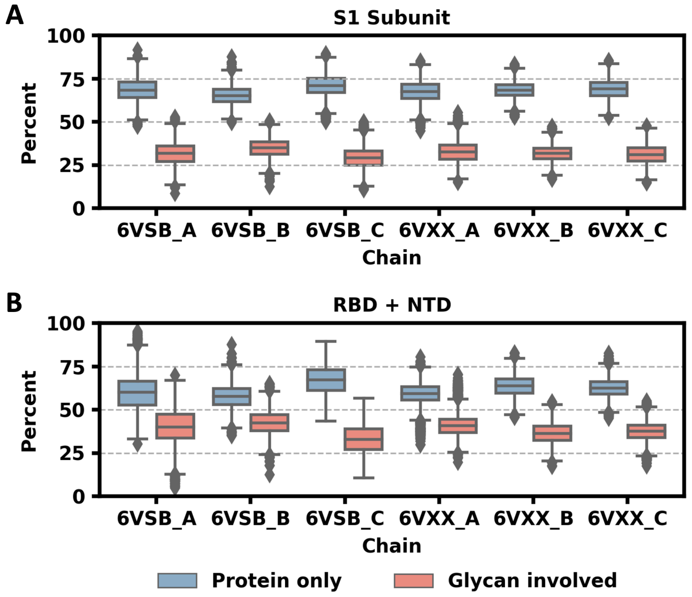

Figure S8. Surface area buried in the interface of S trimer. The total buried surface area split into the portion contributed by protein only and the portion involving the contribution from glycans were calculated for (A) the entire S1 subunit and (B) RBD and NTD only.

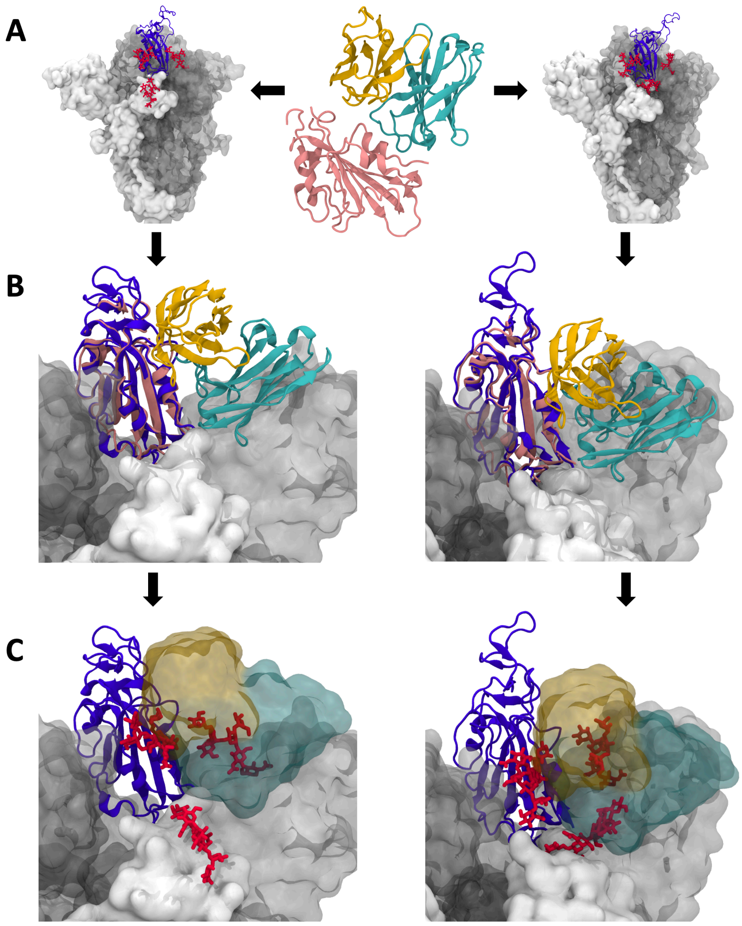

Figure S9. Procedure of measuring the number of glycan atoms clashing with the superimposed antibody. (A) Snapshots are extracted from simulation trajectories. (B) The structure of RBD (pink) in complex with antibody (cyan and yellow) is aligned onto the S trimer structure (RBD shown in blue) by maximizing the overlap between the RBDs from both structures. (C) The number of glycan (red) heavy atoms clashing with the superimposed antibody (less than 1.0 Å) are counted.

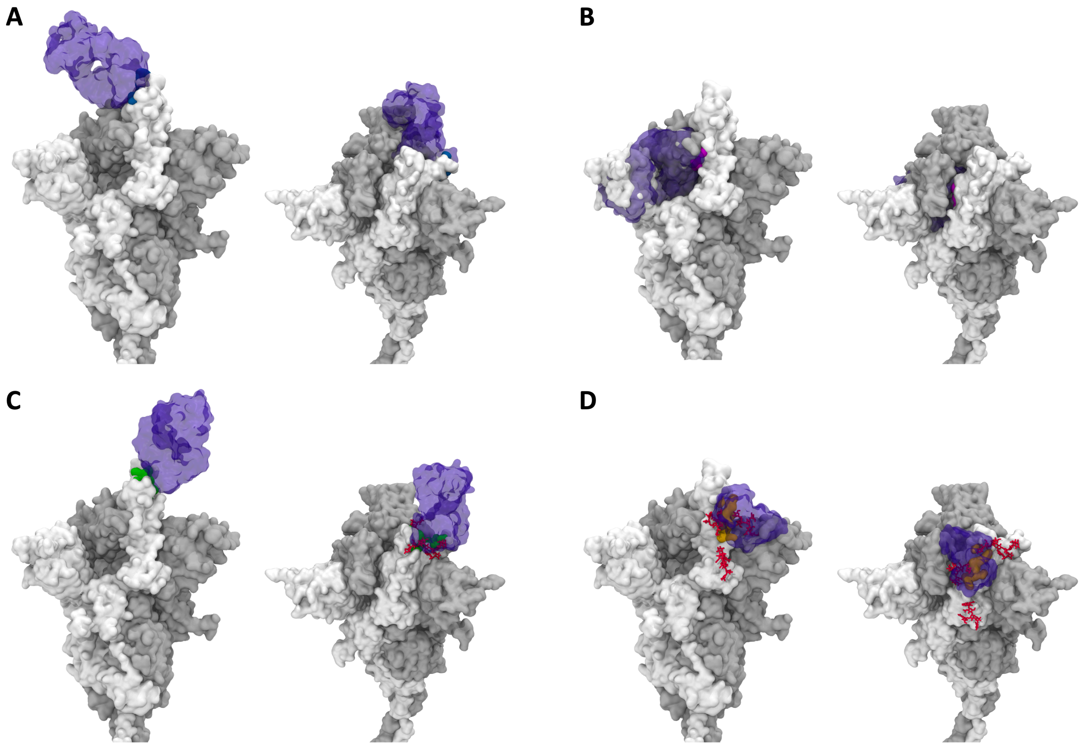

Figure S10. Four antibodies are aligned onto their epitopes in S trimer. (A) B38. (B) CR3022. (C) H11-D4. (D) S309. For each antibody, the RBD in the open state is shown in the side view (left), and the RBD in the closed state is shown in the top view (right). The antibodies are shown in purple, and the colors of corresponding epitopes are the same as those in **Figure 6A**.

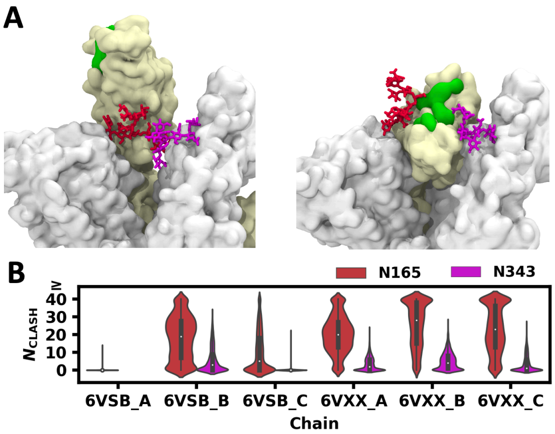

Figure S11. Clash between glycans and superimposed H11-D4 nanobody. (A) H11-D4 epitope (green) in RBD (left: open and right: closed), N165 glycan (red) on the neighboring NTD, and N343 glycan (violet) on the neighboring RBD. (B) Distributions of glycan heavy atom numbers in clash (N_{CLASH}) with the superposed H11-D4 nanobody.

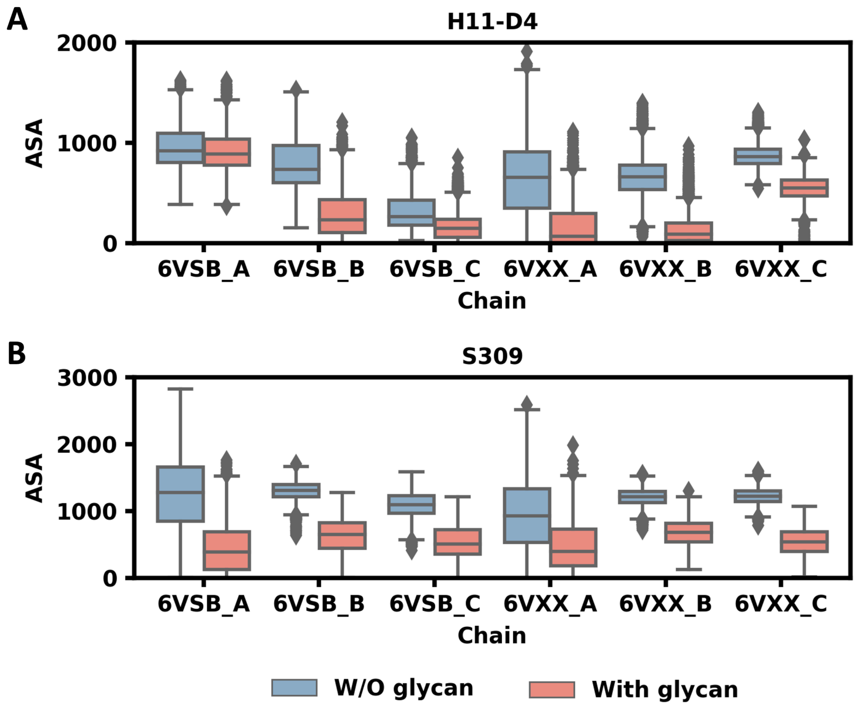

Figure S12. Accessible surface areas of antibody epitopes when glycans are present or removed. The ASA of protein portion in the epitopes of (A) H11-D4 and (B) S309 was first calculated with all glycans removed, and then calculated again with all glycans present. In both cases, a probe radius of 7.2 Å was used.
